# microRNA-Based Biomarkers and the Diagnosis of Alzheimer’s Disease

**DOI:** 10.3389/fneur.2015.00162

**Published:** 2015-07-13

**Authors:** Yuhai Zhao, Surjyadipta Bhattacharjee, Prerna Dua, Peter N. Alexandrov, Walter J. Lukiw

**Affiliations:** ^1^LSU Neuroscience Center Louisiana State University Health Science Center, New Orleans, LA, USA; ^2^Department of Cell Biology and Anatomy, LSU Neuroscience Center Louisiana State University Health Science Center, New Orleans, LA, USA; ^3^Department of Health Information Management, Louisiana State University, Ruston, LA, USA; ^4^Russian Academy of Medical Sciences, Moscow, Russia; ^5^Department of Ophthalmology, LSU Neuroscience Center Louisiana State University Health Science Center, New Orleans, LA, USA; ^6^Department of Neurology, LSU Neuroscience Center Louisiana State University Health Science Center, New Orleans, LA, USA

**Keywords:** aging, Alzheimer’s disease, diagnostic panel, heterogeneity, human biochemical individuality, inflammation, microRNA, prion disease

Alzheimer’s disease (AD) is characterized as a complex, age-related neurological disorder of the human central nervous system (CNS) that involves the progressive mis-regulation of multiple biological pathways at multiple molecular, genetic, epigenetic, neurophysiological, cognitive, and behavioral levels. It has been about 8 years since the first reports of altered microRNA (miRNA) abundance and speciation: (i) in anatomical regions of the brain targeted by the AD process after post-mortem examination, (ii) in blood serum, and (iii) in cerebrospinal fluid (CSF) (1–3). Since then an in depth overview of the peer-reviewed literature has provided no general consensus of what miRNAs are up-or-down regulated in any tissue or biofluid compartment in thousands of AD patients. In this brief “Opinion” paper on “*Biomarkers of Alzheimer’s disease: the present and the future*,” we will highlight the extremely heterogeneous nature of miRNA expression in AD, based on very recent advances in the analysis of miRNA populations in various biofluid compartments compared to normally aging, neurologically normal controls. This work is based against a background of our laboratory’s 24 years of research experience into the structure and function of small, non-coding RNAs in the aging human CNS in health and in age-related neurological disease (4).

First, it is important to appreciate that all forms of dementia due to AD are broadly classified as either early onset (EOAD, under 65 years of age), or late onset (LOAD, over 65 years of age) (5, 6). About ~5% of all AD cases have a genetic component (see below) while the remaining ~95% of all AD cases are of a sporadic (idiopathic) nature or are of unknown origin (5–8). The extremely heterogeneous nature of AD pervades all molecular, genetic, neuropathological and behavioral, mnemonic, and cognitive levels, including the clinical presentation of the disease (6–15). For example, the key *neuropathological markers* of AD include: (i) the progressive deposition of amyloid-beta (Aβ) peptides into dense, insoluble pro-inflammatory senile plaques (SP); (ii) the accumulation of hyperphosphorylated tau into neurofibrillary tangles (NFT); (iii) synaptic atrophy, “pruning” and loss, neuronal degeneration and neuronal cell death; (iv) alterations in the innate-immune response; and (v) the progressive inflammatory neurodegeneration and anatomical targeting of only specific anatomical regions of the brain (1–15). These highly interactive characteristics collectively suggest the participation of multiple pathogenic pathways, and the involvement of multiple deficits in the expression of CNS genes (1–15). Accordingly, this culminates in a remarkably heterogeneous neuropathological scaffold for AD, with significant variations in disease onset, progression, severity of neuropathology, extent of behavioral and cognitive deficits, and memory loss (4–12). To cite one very recent example, a relatively large epidemiological study of AD patient data (*N* = 7815) (12) indicated significant heterogeneity in the first cognitive/behavioral symptomatic “indicator” experienced by AD patients (13–16). In other recent studies, two laboratories have independently reported significant variation in the miRNA-34a-mediated triggering receptor expressed in myeloid/microglial cells-2 (TREM2) down-regulation in an African-American population that further underscores (i) the importance of investigating different ethnic populations for AD epigenetic risk; (ii) intrinsic variance and human *biochemical and genetic individuality*; and (iii) allelic heterogeneity and potentially diverse pathogenic contributory mechanisms to the AD process (sufficient TREM2 is important in the clearance of excessive Aβ peptides from the brain) (9–16). Related to these observations are studies that over the last 15 years have indicated that gene expression patterns at the messenger RNA (mRNA) level, Aβ peptide load, SP and NFT densities and localization, and familial and clinical histories further underscore AD heterogeneity (8–12, 17–20). Indeed, there appears to be intrinsic limitations of useful AD biomarkers because just one biomarker cannot define the mechanism of AD, by nature are associative and/or correlative, and are unable to unequivocally prove disease causality (13–17, 21–23). For example current genome-wide association studies (GWAS), whole-exome and whole-genome sequencing have revealed mutations in excess of 20 genetic loci associated with AD risk (11, 19, 20, 24). Three main genes are involved in EOAD: *amyloid precursor protein* (*APP*), *presenilin 1* (*PSEN1*), and *presenilin 2* (*PSEN2*), while the *apolipoprotein E* (*ApoE*) E4 allele has been found to be a main risk factor for LOAD (1, 17–19, 23). Additionally, recent studies have discovered other genes that might be peripherally involved in AD, including *clusterin* (*CLU*), *complement receptor 1* (*CR1*), *phosphatidylinositol binding clathrin assembly protein* (*PICALM*), *sortilin-related recepto*r (*SORL1*), complement factor H (CFH), the *triggering receptor expressed on myeloid/microglial cells 2* (*TREM2*), and the *cluster of differentiation 33* (*CD33*) gene loci; although not one single case of AD has yet been found to be associated with more than one of these aberrant genetic loci (11, 25). Indeed, most AD cases do not contain any of these mutant genetic “*biomarkers*” (11, 20, 24–26). Further, the persistence of mutations in these genes from birth and throughout life, in contrast to the general development of AD in old age, suggests that multiple age-associated gene regulatory mechanisms must come into play to initiate and drive development and propagation of the AD process, and miRNAs are excellent candidates for these diverse age-related, developmental, and regulatory roles (1–5, 9, 22).

Regarding the rate and variability of cognitive decline in AD, one large recent study did not find evidence supporting a substantial role of the mini-mental status examination (MMSE) as a stand-alone single-administration test in the identification of mild cognitively impaired patients who eventually develop AD, suggesting the need for additional neuropsychological testing and comprehensive biomarker analysis (21–23). Indeed, although AD is the most common form of senile dementia, it can often be challenging to distinguish this insidious and fatal disorder from other equally heterogeneous neurodegenerative disorders, such as frontal temporal dementia, human prion disease [including bovine spongiform encephalopathy (BSE; mad cow disease), Creutzfeldt–Jakob disease, Gerstmann–Sträussler–Scheinker syndrome, and other relatively rare human prion diseases], Huntington’s disease, Lewy Body dementia, Parkinson’s disease, cerebrovascular disease, or vascular (multiple infarct) dementia (16–18, 21–23). Indeed, the diagnostic accuracy of when brain-mediated cognitive deficits actually begin may require a dimensional rather than a categorical classification, and a lifespan rather than aging grouping, and it has been recently suggested that a multidimensional system-vulnerability approach rather than a simple “*hypothetical biomarker*” model of age-associated cognitive decline and dementia may be more useful diagnostically (12, 20). Put another way, AD might be classified not as a discrete disease entity but rather as a “*neurological disconnection syndrome*” (7, 8, 11, 15, 24). This “*neurological disconnection syndrome*” is more broadly defined as an abnormal condition characterized by an established group of variable neurological signs, symptoms, and molecular markers, including miRNA abundance and speciation, that individually possess only limited neuropathological and cognition/behavioral similarities from patient to patient (7–9, 11–18, 21–24).

Further to the concept of AD heterogeneity are the ideas that form the conceptual basis for “*human biochemical and genetic individuality*” (5, 9, 18). These include individual gene sequence variation, gene-based susceptibility to disease and heterogeneity in miRNA abundance and complexity, that may in part drive a general redundancy in gene expression in different human populations (5, 9, 16, 21, 22). Interestingly, these variations may directly impact the genetic evolution of the human species (4, 5, 18–20, 24–26). Much independently derived data support the concept that the genetics, epigenetics, and genome-wide regulatory networks of AD vary considerably among different human populations that possess different genetic and/or environmental backgrounds. Furthermore, despite the fact that genetic factors are inherited and fixed, non-genetic factors, such as (i) environmental or occupational exposures to pesticides, organic solvents, anesthetics, and/or food additives; (ii) pre-existing medical conditions such as cancer, cerebrovascular, and/or cardiovascular disease, depression, diabetes, dyslipidemia, hypertension, traumatic brain injury, older age, female gender, and ApoE status; and (iii) lifestyle factors such as alcohol and coffee consumption, salt, sugar, and cholesterol and fat intake, body mass index, cognitive activity, physical activity, and smoking, are life-style determined and these are known to impact the incidence, development and propagation of AD (18–20, 24–31). Interestingly, certain potentially pathogenic “*pro-inflammatory miRNAs*” of the host are significantly inducible by common microbial and environmental factors such as herpes simplex-1 virus (HSV-1) and naturally occurring elements of the biosphere (such as aluminum oxides that make up almost 9% of the earth’s crust) (32–35).

To make another important point concerning the variable contribution of specific miRNAs to AD, we surveyed the most recently published papers on “*miRNA biomarkers for AD*” using the National Institutes of Health National Library of Medicine website MedLine (www.ncbi.nlm.nih.gov; using the keywords “*Alzheimer’s disease*,” *“miRNA” and “2015*”). The most recent findings of 15 independent labs further support the contention of extremely high miRNA heterogeneity in AD tissue and biofluids (36–50). For example, the last 15 reports of diagnostic markers in AD CSF (36–39; involving miRNA-27a, miRNA-29a, miRNA-191, miRNA-384) and others, AD blood serum (38–46; involving miRNA-107, miRNA-125b, miRNA-128, miRNA-132, miRNA-191, miRNA-206, miRNA-384) and others; “*humanized*” AD cell models (47–50; involving miRNA-125b, miRNA-128, miRNA-138) and others, and several recent reviews (51–55) *provides no common or general consensus of any single miRNA that defines causality for the onset or duration of the AD process*. To further complicate these findings, recent molecular-genetic studies have also shown that even when derived from homogenous source populations, such as pluripotent stem cells, individual cells from those populations exhibit significant differences in gene expression, protein abundance and phenotypic output; here specific families of miRNAs appear to have a deterministic role in reconfiguring the “*pluripotency network*” of individual cells with important downstream functional consequences (47–49, 56, 57).

It is further important to point out exactly what an advanced analytical technique will tell us. For example, most AD researchers would agree that the production of Aβ42 peptides is involved in the AD process. Aβ42 peptides and fragments are generated by a variety of secretases (chiefly α-, β-, and γ-secretases), however, other secretase-like enzymes and enzyme modifiers appear to be involved (5, 8, 14, 25, 31, 58). While RNA-seq and other “next generation sequencing” (NGS) methods will tell us something about the levels of expression of these secretases they would give us no clue about the activity of these secretases in the brain, and their ability to generate Aβ42 or other AD-relevant peptides, which are affected by many other genetic, epigenetic, non-genetic, environmental, and host lifestyle factors. So it is unlikely that RNA-seq, NGS, or other “advanced sequencing methodologies” could give us the entire story of what is going on in AD, although most agree it would give us very valuable insight as to what is happening at the molecular-genetic level, and perhaps be of some value diagnostically.

Lastly, if high-density microarray- and advanced RNA-sequencing based profiles of AD brain or biofluid samples are any indication of AD variability then there are real and significant human population differences in AD onset, incidence, epidemiology, disease course and progression (9, 16, 21, 22, 25, 50, 57). It is unlikely that a single miRNA in the CSF, blood serum, urine, or any other biofluid compartments from multiple human populations will be predictive for AD at any stage of the disease. However, what might be particularly useful for significantly improved AD diagnostics would be a selective, high-density panel of a “*pathogenic and neurodegeneration-associated miRNA family*” that along with other gene expression-based biometrics could more accurately predict the onset of AD-type change. This highly interactive, “*personalized medicin*e” approach – involving a comprehensive evaluation that scores multiple AD deficiencies including miRNA-, mRNA-, and protein-based gene expression alterations, AD-relevant DNA mutations, pro-inflammatory biomarkers (such as C-reactive protein or CRP), and Aβ40- and Aβ42-peptide load in the CSF and blood serum, combined with data from MRI- and PET-based brain imaging, and familial, clinical history, lifestyle, and other factors could be extremely useful in the improved diagnosis of AD susceptibility and development (52–58). These *highly integrated and multidimensional diagnostic approaches* certainly lie within the grasp of current medical technologies – it will just be a matter of improved application, data acquisition and integration of clinical research and healthcare resources to frame a more accurate diagnostic portrait of the “*alleged AD patient*.” Indeed, an equally wide variety of individualistic prevention and “*personalized*” treatment strategies would be required to more effectively address such age-related neurological disorders, including the implementation of combinatorial and/or customized anti-miRNA strategies that have as yet not been considered.

## Conflict of Interest Statement

The authors declare that the research was conducted in the absence of any commercial or financial relationships that could be construed as a potential conflict of interest.

## Abbreviations

miRNA, microRNA.
